# Thyroid autoimmunity and adverse pregnancy outcomes: A multiple center retrospective study

**DOI:** 10.3389/fendo.2023.1081851

**Published:** 2023-02-27

**Authors:** Yun Xu, Hui Chen, Meng Ren, Yu Gao, Kan Sun, Hongshi Wu, Rui Ding, Junhui Wang, Zheqing Li, Dan Liu, Zilian Wang, Li Yan

**Affiliations:** ^1^ Department of Endocrinology, The Sun Yat-sen Memorial Hospital of Sun Yat-sen University, Guangzhou, China; ^2^ Department of Endocrinology, The Sixth Affiliated Hospital, Sun Yat-sen University, Guangzhou, China; ^3^ Department of Obstetrics and Gynecology, The Sun Yat-sen Memorial Hospital of Sun Yat-sen University, Guangzhou, China; ^4^ Department of Obstetrics and Gynecology, The Sixth Affiliated Hospital, Sun Yat-sen University, Guangzhou, China; ^5^ Department of Laboratory, The Sun Yat-sen Memorial Hospital of Sun Yat-sen University, Guangzhou, China; ^6^ Artificial Intelligence Lab and the Big Data Center, The Sun Yat-sen Memorial Hospital of Sun Yat-sen University, Guangzhou, China; ^7^ Network Information Center, The Sixth Affiliated Hospital, Sun Yat-sen University, Guangzhou, China; ^8^ Department of Obstetrics and Gynecology, The First Affiliated Hospital, Sun Yat-sen University, Guangzhou, China

**Keywords:** thyroid autoimmunity, maternal and fetal outcomes, dose dependent effect, pregnancy-induced hypertension, gestational diabetes mellitus, birth weight

## Abstract

**Background:**

The relationship between thyroid autoimmunity (TAI) and adverse pregnancy outcomes is disputable, and their dose-dependent association have not been fully clarified.

**Objective:**

To investigate the association and dose-dependent effect of TAI with multiple maternal and fetal-neonatal complications.

**Methods:**

This study is a multi-center retrospective cohort study based on singleton pregnancies of three medical college hospitals from July 2013 to October 2021. The evolution of thyroid function parameters in TAI and not TAI women were described, throughout pregnancy. The prevalences of maternal and fetal-neonatal complications were compared between the TAI and control group. Logistic regression was performed to study the risk effects and dose-dependent effects of thyroid autoantibodies on pregnancy complications, with adjustment of maternal age, BMI, gravidity, TSH concentrations, FT4 concentrations and history of infertility.

**Results:**

A total of 27408 participants were included in final analysis, with 5342 (19.49%) in the TAI group and 22066 (80.51%) in control group. TSH concentrations was higher in TAI women in baseline and remain higher before the third trimester. Positive thyroid autoantibodies were independently associated with higher risk of pregnancy-induced hypertension (OR: 1.215, 95%CI: 1.026-1.439), gestational diabetes mellitus (OR: 1.088, 95%CI: 1.001-1.183), and neonatal admission to NICU (OR: 1.084, 95%CI: 1.004-1.171). Quantitative analysis showed that increasing TPOAb concentration was correlated with higher probability of pregnancy-induced hypertension, and increasing TGAb concentration was positively correlated with pregnancy-induced hypertension, small for gestational age and NICU admission. Both TPOAb and TGAb concentration were negatively associated with neonatal birthweight.

**Conclusion:**

Thyroid autoimmunity is independently associated with pregnancy-induced hypertension, gestational diabetes mellitus, neonatal lower birthweight and admission to NICU. Dose-dependent association were found between TPOAb and pregnancy-induced hypertension, and between TGAb and pregnancy-induced hypertension, small for gestational age and NICU admission.

## Introduction

Thyroid autoimmunity (TAI) is defined as the presence of antibodies to thyroperoxidase (TPOAb) or thyroglobulin (TGAb) (1). The prevalence of TAI is 5-15% in reproductive aged women (2) and even higher in pregnant women (5-20%) (3–6).

Compared to thyroid dysfunction, the impact of TAI on pregnancy might be underestimated. Available evidence predominantly links the adverse pregnancy outcomes in TAI women to hypothyroidism (7), and clinical guidelines recommended levothyroxine supplement as the only treatment method (8). However, euthyroidism was found in the majority of (75%) pregnant women with TAI (9). Increasing studies showed that the association of TAI with miscarriage and preterm birth remained significantly after adjustment for thyroid dysfunction (10), and the pregnancy outcomes has not found to be improved by thyroid hormone replacement. Dhillon-Smith reported that adverse neonatal outcomes were not different after levothyroxine supplement in TPOAb positive women (11). Similarly, a recent multicenter RCT (T4LIFE trial) showed that, supplement of levothyroxine did not improve pregnancy outcomes in euthyroid TAI women (12). These results indicated that TAI in itself may induce adverse pregnant outcomes besides *via* mediating thyroid destruction.

Although with different results, the association between TAI and recurrent miscarriage and preterm birth were identified by prospective cohort studies and meta-analysis (10, 13, 14). Recently, increasing studies focus on the impact of TAI on other pregnancy complications, including pregnancy-induced hypertension (15, 16), gestational diabetes (17, 18), and adverse fetal-neonatal outcomes (16, 19, 20), but the relationship had not been fully clarified to draw any conclusions. The possible reason of this is that most of the studies included small sample and only focused on single outcome and without adjustment of confounders. In addition, as the reflection of thyroid autoimmunity process, there is a dose-dependent association of thyroid autoantibodies with TSH and free thyroxine level in pregnant women (21), however, study assessing dose-dependent association of thyroid autoantibodies with adverse pregnancy outcomes were seldom to date. To accessing their dose-dependent associations should provide insights toward distinguishing low-risk from high-risk individuals and optimizing clinical decision-making strategies.

Therefore, based on our large multicenter cohort, the purpose of the present study is to verified the association, as well as the dose-dependent effect of TAI with various maternal and fetal-neonatal complications.

## Methods

The study was registered in Chinese Clinical Trial Registry (ChiCTR2200064466) and was approved by the ethical committees of Sun Yat-sen Memorial Hospital of Sun Yat-sen University with a waiver of informed consent (SYSEC-KY-KS-2020-200).

### Study design and participants

This is an observational cohort study based on the electronic medical record in three college hospitals, the Sun Yat-sen Memorial Hospital, the First Affiliated Hospital, and the Sixth Affiliated Hospital of Sun Yat-sen University. Data of pregnant women delivered in the Department of Obstetrics in these three hospitals from July 2013 to October 2021 were included for primary screening base on the following criteria: (1) with thyroid autoantibodies (TPOAb or TGAb) results obtained in the first and second trimester or within one year before pregnancy; (2) 18-55 years old; (3) with complete records of pregnant outcomes. Participants with the following criteria were excluded: (1) with medical history of thyroid diseases before pregnancy (i.e. hyperthyroidism, thyroid cancer, surgical history on thyroid, and pituitary diseases); (2) termination due to fetal abnormality, chromosomal abnormality, maternal chronic diseases or personal reasons; (3) with multiple pregnancy.

### Collection of information

From the medical records, we collected basial and gestational characteristics (age, height, weight at admission for delivery, gestational age, gravidity, parity, delivery mode, past medical history and family medical history), gestational complications and adverse outcomes (preterm birth, gestational diabetes mellitus, pregnancy-induced hypertension, postpartum hemorrhage and premature rupture of membrane), neonates (birthweight, Apgar score, need for intensive neonatal care, neonatal death). For each participant, the serum concentrations of TPOAb, TGAb, thyroid stimulating hormone (TSH), free triiodothyronine (FT3), free thyroxine (FT4) during pregnancy were all obtained and the mean gestational weeks of the first blood sample detection were 15.4 ± 6.4 gestational weeks. These thyroid parameters were examined by chemiluminescent immunoassay, and the detail of laboratory measurements from each hospital was summarized in **Supplementary Table S1**.

### Definition of TAI and pregnancy complications

TPOAb and TGAb positivity was defined according to the cutoffs provided by the manufacturers. Participants with positive results of either TPOAb or TGAb in the first or second trimester during pregnancy, or within one year before pregnancy were included in the TAI group, while those with both TPOAb and TGAb in normal range were in control group. The definition of thyroid function parameters was according to the reference range for each trimester of each hospital.

Adverse pregnancy outcomes comprised maternal and fetal-neonatal outcomes and the definition were according to the practical guidelines of each disease. Preterm birth (PTB) was defined as termination of pregnancy between 28 and <37 gestational weeks. Gestational diabetes mellitus (GDM) diagnosed *via* 75g glucose OGTT test when one or more plasma glucose (PG) meet or exceed the thresholds: fasting PG 5.1 mmol/L, 1h-PG 10.0 mmol/L, and 2h-PG 8.5 mmol/L, according to the International Association of Diabetes and Pregnancy Study Groups-2010 guidelines (22), and women diagnosed with overt diabetes before pregnancy were excluded. Pregnancy-induced hypertension (PIH) was defined as maternal blood pressure exceeding 140/90 mmHg induced by pregnancy after 20 weeks’ gestation with or without proteinuria and edema (23). Premature rupture of membrane (PROM) was defined as abruption of the amniotic sac before labor onset. Postpartum hemorrhage was defined as a cumulative blood loss of greater than or equal to 500mL for vaginal delivery or 1,000 mL for cesarean. Large for gestational age (LGA) and small for gestational age (SGA) which determined according to the National Institute of Child Health and Human Development (NICHD) standard for Asian population (24). Macrosomia was defined as neonatal birthweight heavier than 4000 grams and Apgar Score ≤7 at 1 to 10 minute after born were defined as Low Apgar Score.

### Study outcomes and data analysis

The primary outcome was the association between TAI and various pregnancy complications. Maternal outcomes including PTB, PIH, GDM, PROM and postpartum hemorrhage. Fetal-neonatal outcomes including need for intensive neonatal care, Low Apgar Score, macrosomia, LGA and SGA.

Research data was analyzed by Python 3.8. Continuous variables were reported with mean and standard deviation, and categorical variables were reported with number and percentage. The difference between group were compared by Chi-squared test for categorical variables. Independent Student’s t test and Mann-Whitney test were applied to compared continuous variables with normally and normally distributions, respectively. Two-sided *P* values less than 0.05 were considered statistically significant.

The association between TAI and pregnancy complications were firstly assessed by chi-squared test. Then, multiple logistic regression analysis was applied to adjusted confounders and build multiple models: (1) Model1: adjusted for maternal age and BMI; (2) Model2: Model 1+gravidity; (3) Model3: Model 2+TSH and FT4 concentration; (4) Model4: Model3 + history of infertility. For the risk of GDM and PIH, family medical history of diabetes and hypertension were considered, respectively. For the risk of preterm birth, history of recurrent miscarriage was considered. The concentrations of TPOAb and TGAb were first compared in participants with and without each maternal and fetal-neonatal complications, and those complications with TPOAb or TGAb difference were further studied for their dose-dependent effect. The associations between pregnancy complications with TPOAb or TGAb concentrations were analyzed *via* logistic regression with the same adjustment above respectively. The association between neonatal birthweight and TPOAb or TGAb concentrations were analyzed by ANCOVA with adjustment of confounders in the Model 4.

## Results

### Basal characteristics of participants

**Figure 1:**
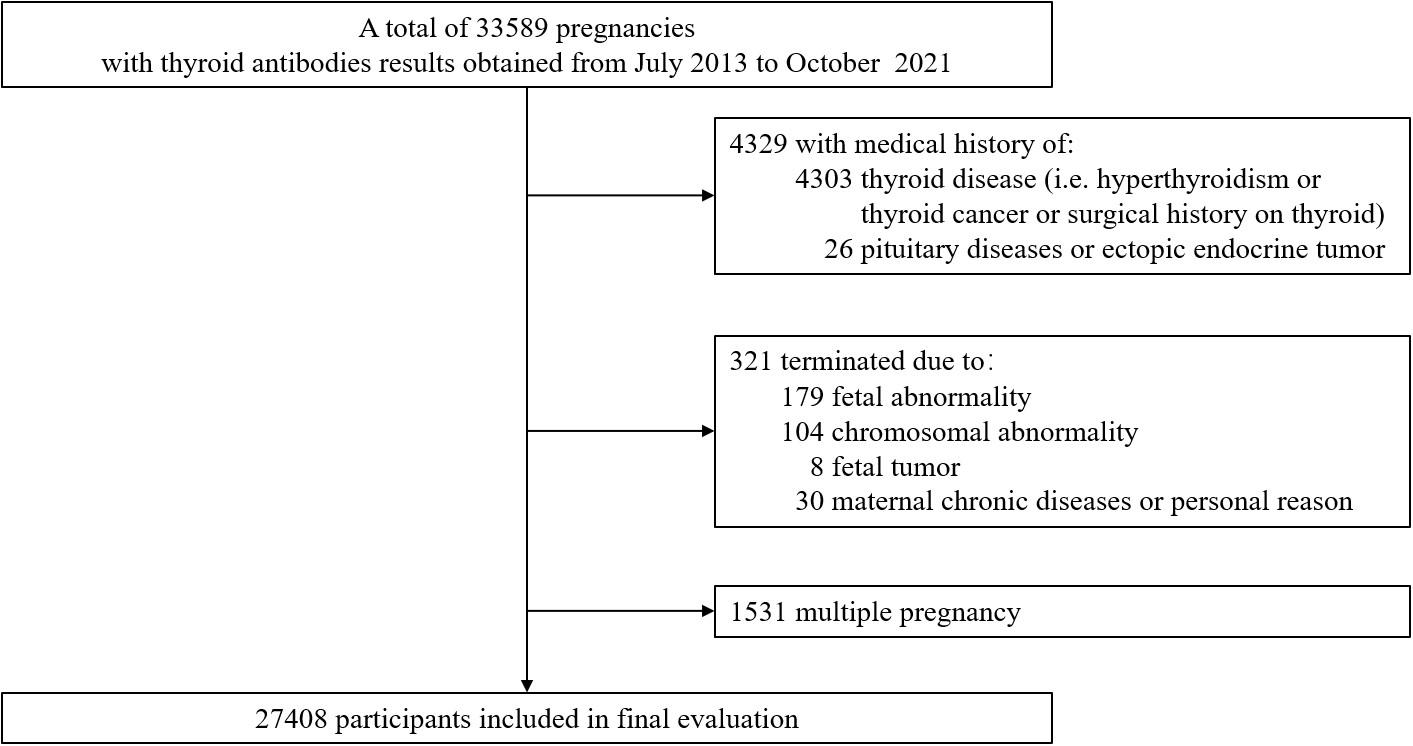
Flow chart of research population selection.

A total of 33589 pregnancies records met the inclusion criteria. Of these, a total of 6181 were excluded due to maternal thyroid diseases before pregnancy (n=4303), pituitary diseases or ectopic endocrine tumor (n=26), terminated due to fetal abnormality (n=179), chromosomal abnormality (n=104), fetal tumor (n=8), maternal chronic diseases or personal reason (n=30), and multiple pregnancy (n=1531). The final study cohort comprised 27408 pregnancies. **Figure 1** demonstrated the flow chart of data selection.

Of all 27408 participants, 5342 (19.5%) pregnancies which have at least one record showed positive TPOAb or TGAb (TAI group) while 22066 (80.5%) pregnant women with negative TPOAb and TGAb results in all detected records (control group). In the TAI group, 2641(49.4%) were only TPOAb positive, 917 (17.2%) were only TGAb positive, and 1784 (33.4%) were positive for both antibodies. In TAI groups, the mean concentration of TSH first tested in pregnant period was higher (1.94 ± 2.42μIU/ml *vs.* 1.43 ± 1.01μIU/ml, *P <*0.001), and ratio of pregnancies with TSH concentration between 2.5 and 4μIU/ml (17.8% *vs.* 10.3%, *P <*0.001) or exceeded 4.0μIU/ml (7.0% *vs.* 2.0%, *P <*0.001) were increased significantly (**Table 1**). That means pregnant women in TAI group have lower ratio of meeting treatment target and higher probability of subclinical hypothyroidism.

**Table 1:** Baseline characteristics of research population.

Characteristics	TAI Positive	TAI Negative	*P* value
**Number**	5342 (19.5%)	22066 (80.5%)	
**Maternal Age (years)**	32.1 ± 4.6	31.7 ± 4.6	**< 0.001**
**BMI (kg/m^2^)**	26.1 ± 3.0	26.1 ± 3.0	0.331
Gravidity			
* Primigravida*	1682 (31.5%)	7913 (35.9%)	**< 0.001**
* Multigravida*	3653 (68.5%)	14125 (64.1%)	
Parity			
* Nullipara*	3185 (60.4%)	12277 (56.1%)	**< 0.001**
* Multipara*	2090 (39.6%)	9616 (43.9%)	
History of Recurrent Miscarriage			
* Yes*	850 (16.0%)	1224 (5.6%)	**< 0.001**
* No*	4446 (84.0%)	20633 (94.4%)	
History of Infertility			
* Yes*	792 (14.8*%*)	2575 (11.7*%*)	**< 0.001**
* No*	4550 (85.2*%*)	19491 (88.3*%*)	
Family History of Diabetes			
* Yes*	395 (7.4*%*)	1680 (7.6*%*)	0.607
* No*	4947 (92.6*%*)	20386 (92.4*%*)	
Family History of Hypertension			
* Yes*	726 (13.6*%*)	3197 (14.5*%*)	0.097
* No*	4616 (86.4*%*)	18869 (85.5*%*)	
**Free T4 Concentrations (pmol/L)**	12.58 ± 3.80	11.88 ± 3.30	**< 0.001**
**TSH Concentrations (μIU/ml)**	1.94 ± 2.42	1.43 ± 1.01	**< 0.001**
**TSH Concentrations Classification**			**< 0.001**
* ≤ 2.5 μIU/ml*	3973 (75.2*%*)	19143 (87.7*%*)	
* 2.5-4.0 μIU/ml*	941 (17.8*%*)	2244 (10.3*%*)	
* > 4.0 μIU/ml*	371 (7.0 ***%***)	451 (2.0 ***%***)	

P values <0.05 were shown in bold.

The basal characteristics of the participants were summarized in **Table 1**. Pregnant women in the TAI group were older (32.1 ± 4.6 years *vs*. 31.7 ± 4.6 years, *P <*0.001), with more gravidity (multigravida: 68.5% *vs*. 64.1%, *P <*0.001), but less parity (multipara: 39.6% *vs*. 43.9%, *P <*0.001). Participants in the TAI group were with higher proportion of recurrent miscarriage history (16.0% *vs*. 5.6%, *P <*0.001) and infertility history (14.8% *vs*. 11.7%, *P<*0.001). The gestational age at terminate was mild lower in TAI group (267 ± 25 days *vs*. 270 ± 21 days, *P <*0.001) and the neonates born to mother with TAI were with lower birthweight (3089.1 ± 457.3 gram *vs*. 3139.4 ± 453.0 gram, *P <*0.001). The differences of BMI, family histories of diabetes and hypertension were not statistically significant between groups.

### Evolution of maternal thyroid parameters during pregnancy

**Figure 2:**
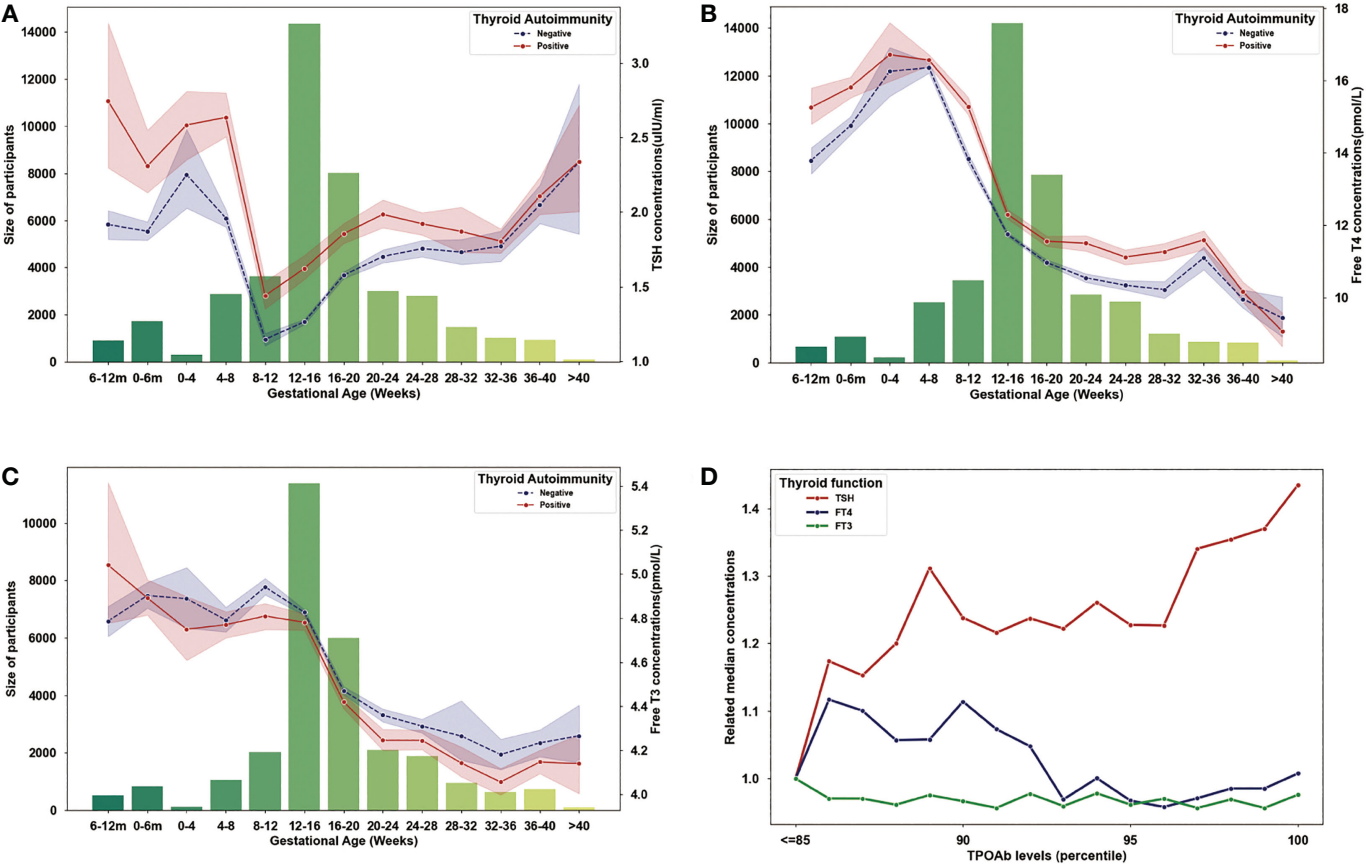
Evolution of maternal thyroid parameters during pregnancy. **(A)** Variation of TSH concentrations (lines) and sample size (bars) in each gestational month (the first and second bars represent 6-12 months and 0-6 months before pregnancy). **(B)** Variation of Free T4 concentrations (lines) and sample size (bars) in each gestational month (the first and second bars represent 6-12 months and 0-6 months before pregnancy). **(C)** Variation of Free T3 concentrations (lines) and sample size (bars) in each gestational month (the first and second bars represent 6-12 months and 0-6 months before pregnancy). **(D)** Evolution of TSH, Free T4 and Free T3 level by TPOAb concentrations (related to the concentrations of TSH, Free T4 and Free T3 in cases with TPOAb concentration lower or equal to the 85 percentile).

To describe the evolution of thyroid function parameters in TAI and not TAI women, throughout pregnancy. We obtained all results of TSH, FT4, and FT3 during pregnant period. In both TAI and control group, the concentration of TSH decreased in the first eight to twelve gestational weeks and gradually increase from then on (**Figure 2A**), however, FT4 and FT3 both continuously decreased during the pregnant period (**Figure 2B, C**). Compared to control group, the concentrations of TSH in the TAI group were significantly higher in both first and second trimester (first trimester: 1.91 ± 1.05 μIU/ml *vs*. 1.31 ± 1.05 μIU/ml, *P*<0.001; second trimester: 1.86 ± 1.54 μIU/ml *vs*. 1.56 ± 1.04 μIU/ml, *P*<0.001; third trimester: 1.92 ± 1.71 μIU/ml *vs*. 1.85 ± 1.29 μIU/ml, *P*=0.155) (**Figure 2A**), and the concentrations of FT3 were lower during period (first trimester: 4.81 ± 0.70 μIU/ml *vs*. 4.88 ± 0.69 μIU/ml, *P*<0.001; second trimester: 4.41 ± 0.78 μIU/ml *vs*. 4.50 ± 0.63 μIU/ml, *P*<0.001; third trimester: 4.12 ± 0.62 μIU/ml *vs*. 4.23 ± 1.08 μIU/ml, *P*=0.001) (**Figure 2C**) and FT4 were higher (first trimester: 14.37 ± 4.00 μIU/ml *vs*. 12.76 ± 3.56 μIU/ml, *P*<0.001; second trimester: 11.67 ± 3.36 μIU/ml *vs*. 11.06 ± 2.94 μIU/ml, *P*<0.001; third trimester: 11.01 ± 3.01 μIU/ml *vs*. 10.36 ± 2.96 μIU/ml, *P*<0.001) (**Figure 2B**).

Then, we described the variation of thyroid function indicators with TPOAb concentration increasing. Because the reference ranges were different among kits, we used population-based percentiles of TPOAb concentrations for each kit to investigate the quantitative effect on thyroid function, and the 85 percentiles represent positive results of all kits. As seen in **Figure 2D**, we found TSH increased in the cases with TPOAb concentrations higher than 85 percentiles, and FT4 decrease with TPOAb concentrations in the cases with TPOAb concentrations higher than 90 percentiles, but FT3 did not have dose–response effect of TPOAb.

### Thyroid autoimmunity and pregnancy complications

The prevalence of pregnancy complications was compared between TAI and control group (**Table 2**). Pregnant women in the TAI group were with higher proportion of PIH (4.06% *vs*. 3.45%, *P*=0.037), GDM (20.39% *vs*. 18.45%, *P*=0.001) and PTB (8.95% *vs*. 7.56%, *P*=0.001). Neonates born to mothers in the TAI group were with less macrosomia (1.55% *vs*. 2.20%, *P*=0.003) and LGA (4.04% *vs*. 4.77%, *P*=0.045), but more incidence of NICU admission (24.11% *vs*. 21.77%, *P*<0.001). In addition, no difference was found in other outcomes.

**Table 2:** Proportions of pregnant complications between groups.

	TAI Positive	TAI Negative	*P* value
Maternal Outcomes			
Pregnancy Induce Hypertension	212 (4.06%)	748 (3.45%)	**0.037**
Gestational Diabetes Mellitus	1059 (20.39%)	3974 (18.45%)	**0.001**
Preterm Birth	469 (8.95%)	1645 (7.56%)	**0.001**
Premature Rupture of Membrane	1060 (19.84%)	4454 (20.19%)	0.589
Postpartum Hemorrhage	377 (7.06%)	1711 (7.75%)	0.090
Fetal-neonatal Outcomes			
Neonatal Outcome			**< 0.001**
*Normal*	3962 (75.74%)	16965 (78.08%)	
*NICU Admission*	1261 (24.11%)	4730 (21.77%)	
*Neonatal Death*	8 (0.15%)	33 (0.15%)	
Apgar Score			0.546
0-3	25 (0.48%)	94 (0.43%)	
4-7	103 (1.97%)	477 (2.20%)	
*8-10*	5096 (97.55%)	21131 (97.37%)	
Macrosomia	83 (1.55%)	486 (2.20%)	**0.003**
Large for Gestational Age	174 (4.04%)	844 (4.77%)	**0.045**
Small for Gestational Age	545 (11.64%)	2036 (10.77)	0.094

Data are presented as mean ± SD or n (%). P values <0.05 were shown in bold.

Then, we investigated the association between TAI and complications in logistic regression (**Table 3**). After adjusted for maternal age, BMI, gravidity, TSH and F4 concentrations, and history of infertility, TAI were positively associated with PIH (OR: 1.206, 95%CI: 1.019-1.428, *P*=0.030), GDM (OR: 1.088, 95%CI: 1.001-1.183, *P*=0.046), PTB (OR: 1.129, 95%CI: 1.001-1.273, *P*=0.048) and admission of NICU (OR: 1.084, 95%CI: 1.004-1.171, *P*=0.040), and negatively associated with macrosomia (OR: 0.768, 95%CI: 0.599-0.985, *P*=0.038) and large for gestational age(OR: 0.833, 95%CI: 0.695-0.999, *P*=0.049). The results of PIH and GDM were remain statistically significant after additional adjustment by family history of hypertension and diabetes respectively (PIH: OR: 1.215, 95% CI 1.026-1.439, *P*=0.024; GDM: OR: 1.088, 95% CI 1.001-1.183, *P*=0.048). The result of preterm birth was not statistically significant after additional adjustment by history of recurrent miscarriage (OR=1.082, 95% CI 0.958-1.222, *P*=0.205).

**Table 3:** Logistic regression of thyroid autoimmunity and pregnant complications.

	Model 1		Model 2		Model 3		Model 4	
	OR (95% CI)	*P*	OR (95% CI)	*P*	OR (95% CI)	*P*	OR (95% CI)	*P*
Maternal Outcomes								
Pregnancy Induce Hypertension	**1.186 (1.005-1.400)**	**0.043**	**1.195 (1.012-1.410)**	**0.036**	**1.215 (1.026-1.438)**	**0.024**	**1.206 (1.019-1.428)**	**0.030** ^a^
Gestational Diabetes Mellitus	**1.090 (1.004-1.183)**	**0.039**	**1.089 (1.003-1.181)**	**0.042**	**1.093 (1.006-1.188)**	**0.036**	**1.088 (1.001-1.183)**	**0.046** ^b^
Preterm Birth	**1.144 (1.017-1.286)**	**0.025**	**1.138 (1.012-1.280)**	**0.031**	**1.140 (1.011-1.285)**	**0.032**	**1.129 (1.001-1.273)**	**0.048** ^c^
Premature Rupture of Membrane	1.022 (0.944-1.105)	0.596	1.032 (0.954-1.117)	0.431	1.055 (0.974-1.144)	0.188	1.056 (0.975-1.144)	0.183
Postpartum Hemorrhage	0.920 (0.815-1.038)	0.176	0.923 (0.818-1.042)	0.195	0.926 (0.819-1.046)	0.217	0.920 (0.814-1.041)	0.186
Fetal-neonatal Outcomes								
NICU Admission	**1.134 (1.052-1.223)**	**0.001**	**1.140 (1.057-1.230)**	**0.001**	**1.091 (1.010-1.179)**	**0.027**	**1.084 (1.004-1.171)**	**0.040**
Low Apgar Score	0.902 (0.731-1.113)	0.336	0.904 (0.733-1.116)	0.347	0.925 (0.747-1.144)	0.471	0.920 (0.744-1.139)	0.446
Macrosomia	**0.735 (0.575-0.940)**	**0.014**	**0.732 (0.572-0.936)**	**0.013**	**0.769 (0.599-0.986)**	**0.038**	**0.768 (0.599-0.985)**	**0.038**
Large for Gestational Age	**0.813 (0.680-0.972)**	**0.023**	**0.804 (0.672-0.961)**	**0.016**	**0.834 (0.696-1.000)**	0.050	**0.833 (0.695-0.999)**	**0.049**
Small for Gestational Age	1.096 (0.984-1.219)	0.095	1.111 (0.998-1.237)	0.054	1.098 (0.985-1.225)	0.092	1.097 (0.984-1.223)	0.096

Model 1: Adjusted for Age and BMI; Model 2: Model 1+ Gravidity; Model 3: Model 2+ TSH and FT4; Model 4: Model 3+ history of infertility.

a The association between TAI and pregnancy induce hypertension were further adjusted by the confounders in Model 4 and family history of hypertension, and the OR is 1.215(95% CI 1.026-1.439), P=0.024.

b The association between TAI and gestational diabetes mellitus were further adjusted by the confounders in Model 4 and family history of diabetes, and the OR is 1.088(95% CI 1.001-1.183), P=0.048.

c The association between TAI and preterm birth were further adjusted by the confounders in Model 4 and history of recurrent miscarriage, and the OR is 1.082(95% CI 0.958-1.222), P=0.205.

Data are presented as n (%). P values <0.05 were shown in bold.

### Quantitative association between thyroid autoantibodies and complications

To investigate the quantitative association with complications, we first analyze the concentrations (as percentiles) of TPOAb and TGAb in participants with and without each maternal and fetal-neonatal complications. As shown in **Supplementary Table S2**, TPOAb concentrations were higher in pregnant women with pregnancy induced hypertension, gestational diabetes mellitus and preterm birth, and lower in women who given birth to macrosomia. TGAb concentrations were higher in pregnant women with pregnancy induced hypertension, preterm birth, and in mother who given birth to babies with small for gestational age or needed therapy in NICU (**Supplementary Table S2**).

**Figure 3:**
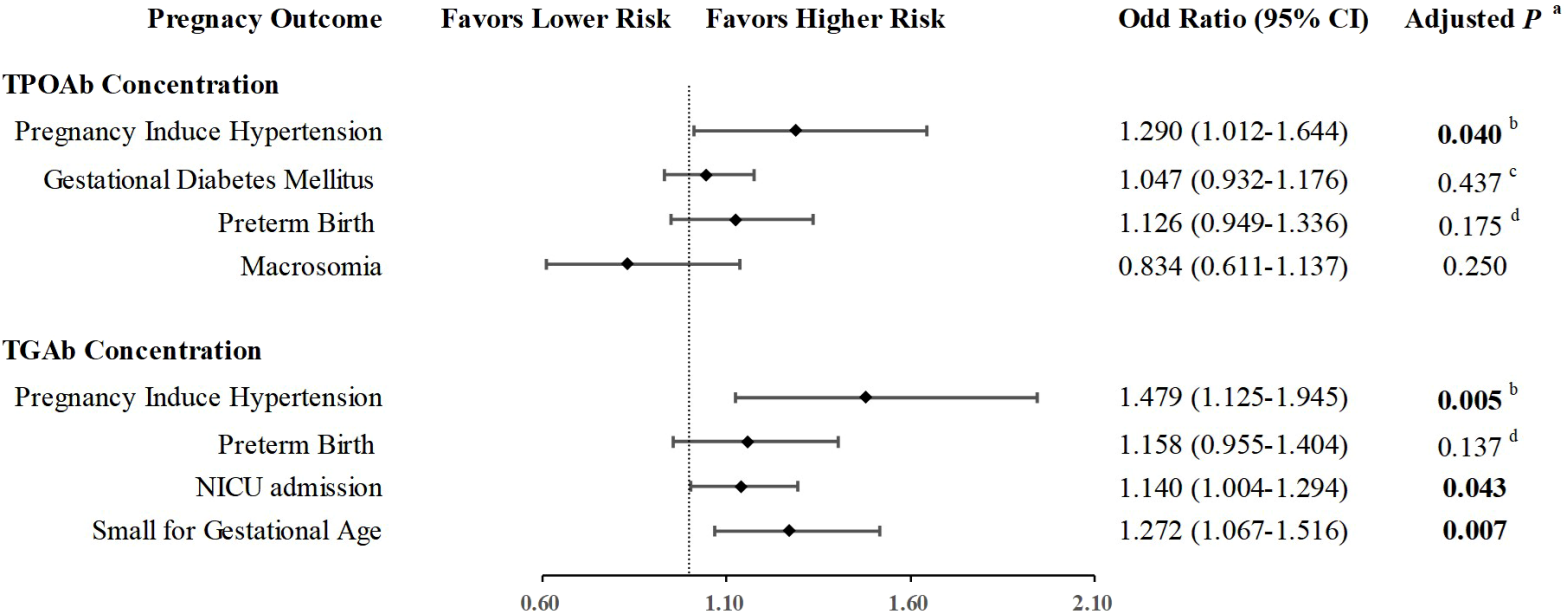
Quantitative association of thyroid autoimmunity and pregnant complications. ^a^Adjusted for Age, BMI, Gravidity, TSH level, Free T4 level, and history of infertility. ^b^Adjusted for Age, BMI, Gravidity, TSH level, Free T4 level, history of infertility, and family history of hypertension. ^c^Adjusted for Age, BMI, Gravidity, TSH level, Free T4 level, history of infertility, and family history of diabetes. ^d^ Adjusted for Age, BMI, Gravidity, TSH level, Free T4 level, history of infertility, and history of recurrent miscarriage.

Then, the associations between TPOAb concentrations and pregnancy induced hypertension, gestational diabetes mellitus, preterm birth, and macrosomia, as well as the associations between TGAb concentrations and pregnancy induced hypertension, preterm birth, SGA and NICU admission were analyzed *via* logistic regression (**Figure 3**). After adjusted for maternal age, BMI, gravidity, TSH and FT4 concentrations, and history of infertility, the probability of PIH rise in paralleled with increasing TPOAb (OR: 1.284, 95%CI: 1.008-1.635, *P*=0.043) and TGAb concentration (OR: 1.450, 95%CI: 1.104-1.905, *P*=0.008) respectively. The effect remains significant even after adjustment with family history of hypertension (TPOAb: OR: 1.290, 95%CI: 1.012-1.644, *P*=0.040; TGAb: OR: 1.479, 95%CI: 1.125-1.945, *P*=0.005). In addition, increasing TGAb concentration were associated with rising positivity of SGA (OR: 1.272, 95%CI: 1.067-1.516, *P*=0.007) and NICU admission (OR: 1.140, 95%CI: 1.004-1.294, *P*=0.043).

**Figure 4:**
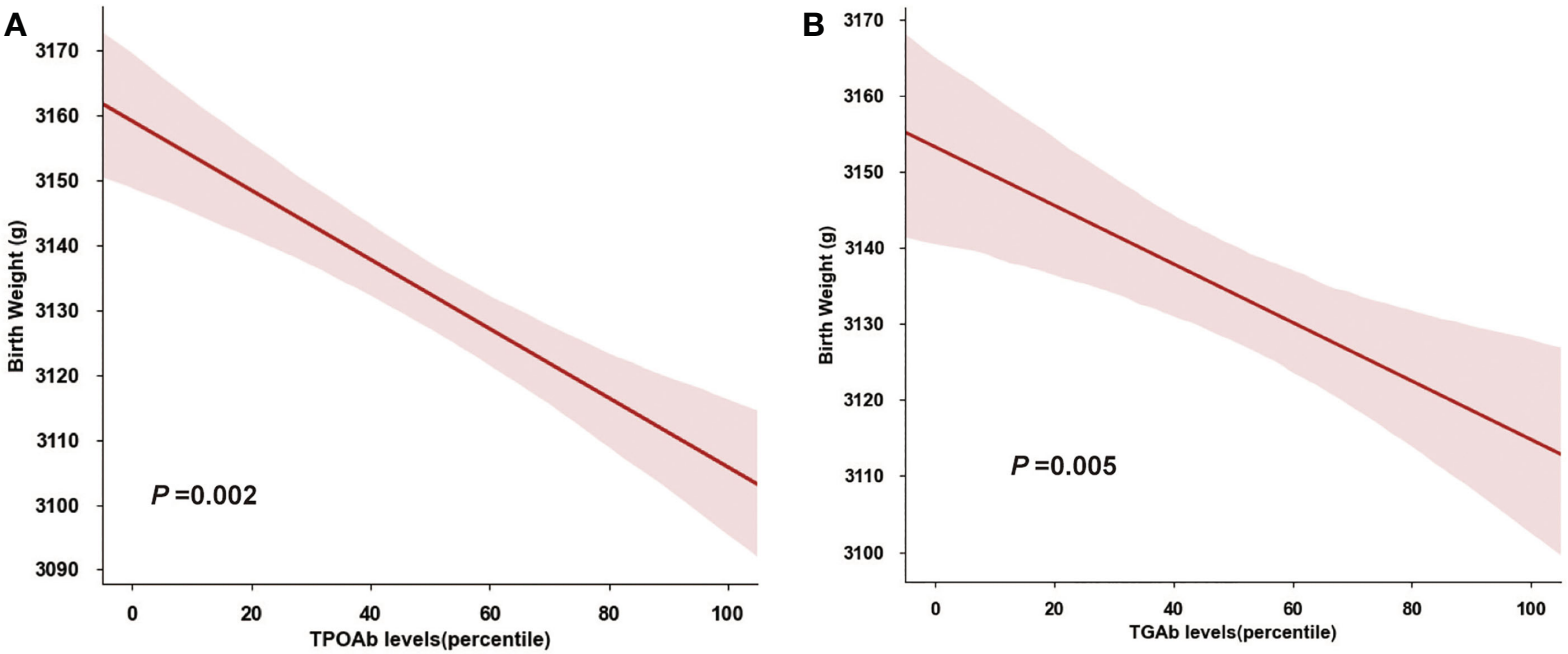
Quantitative Association of Birthweight and Thyroid Autoantibody. **(A)** Birthweight decreased with increasing TPOAb concentrations (percentiles). **(B)** Birthweight decreased with increasing TGAb concentrations (percentiles).

We also performed an ANCOVA analysis on the association between thyroid autoantibodies and birthweight (**Figure 4A, B**), and found that neonatal birthweight was decreased with TPOAb and TGAb level elevating (TPOAb: *P*=0.002; TGAb, *P*=0.005).

## Discussion

In this large multi-center cohort study comprised 27408 pregnancies, we investigated the impact of TAI on multiple types of gestational complications and adverse pregnant outcomes. We verified that pregnant women with positive thyroid autoantibodies had increased risks of pregnancy-induced hypertension, gestational diabetes mellitus, and neonatal admission of NICU. Besides, we found the dose-dependent effect of TPOAb concentrations on pregnancy-induced hypertension, and TGAb concentration on pregnancy-induced hypertension, small for gestational age, neonatal birthweight and NICU admission. In addition, we describe the evolution of maternal serum TSH, FT4 and FT3 and determined their dose-dependent association with thyroid autoantibody. This result may provide insights toward better understanding the difference between TAI women and their control individuals.

Our present study showed that TAI in pregnancy was associated with increasing risk of PIH by approximate 20%, independent from thyroid function. Although hypertensive disorder is increased in patients with hypothyroidism (25–27), the association of thyroid autoimmunity with pregnancy-induced hypertension is disputable. Our result supported the finding of the recent Ma’anshan cohort (the MABC study) in Chinese pregnant women that TAI was positively associated with gestational hypertension (15). Saki’s study also observed higher systolic blood pressure and a higher incidence of preeclampsia in pregnant women with either TPOAb or TGAb positive pregnant women (28). Still, there were some other studies shown no association between TAI and PIH, potentially because a relatively low incidence of individuals with both TAI and PIH in general pregnancies and small sample size did not have the power to find the association. Additionally, some studies in which thyroid autoantibodies were tested in the third trimester did not show association with PIH (20, 29), potentially because that, thyroid autoantibody levels fall and reaching its nadir in the third trimester (6) and the impact of thyroid autoantibodies on blood pressure may exist before this period. Family history contribute a lot to PIH, but previous study did not consider it as confounder. After adjusted for parameters including hypertension family history, we determined the association between thyroid autoantibodies and PIH, more importantly, with a dose-dependent manner. The quantitative association of pregnancy outcomes with thyroid antibodies, especially TGAb were seldom reported, and our result provide evidence that the probability of PIH rise by increasing TPOAb and TGAb level respectively. Taken together, the present results indicated the importance of monitoring blood pressure during pregnancy period in women with TAI, both TPOAb and TGAb positive in early pregnant period should be concerned, especially who with high concentration.

Although increasing studies show the relationship between TAI and GDM (17, 18, 30), it remains not wildly concerned in clinical practice. Thyroid dysfunction may impact the regulation glucose metabolism and increase risk of GDM (17) and current evidence predominantly links it to hypothyroidism. However, Huang’s study found that TAI in itself has higher risk of GDM (31). In the present study, we also identified TAI as independent risk factor of GDM in pregnant women, the result was not impacted by thyroid function even after adjustment with family history of diabetes. That means blood glucose monitoring is important for TAI pregnant women no matter thyroid dysfunction or not. The attention to sugar metabolism even should last for postpartum by reason of Tang’s study also reported that TAI may increase the risk of diabetes mellitus after pregnancy (17). Similar to PIH, studies on GDM in which thyroid autoantibodies positive in the third trimester did not observed a significantly association. Notably, the majority of studies shown positive association between TAI and GDM were perform in Asian population. The underlining mechanism remain uncleared and it needs further investigation.

The association between TAI and preterm birth has been found in previous studies (14, 32–35). In the present study, we found a higher proportion of preterm birth in pregnant women with TAI, and the association between TAI and preterm birth was statistically significant after adjusted for maternal age, BMI, gravidity, TSH and F4 concentrations, and history of infertility. However, after adding history of recurrent miscarriage as confounder, the association between TAI and preterm birth was not found. This result indicated that the impact of TAI on preterm birth needed further investigation.

The impact of thyroid autoantibodies on other outcomes of the developing fetus and neonates is far from elucidated. Although the risk of SGA in TAI group did not meet statistical significance, its probability rises by increasing TGAb level. In addition, neonates born to TAI women tend to be with lower birthweight and higher risk of NICU admission. This result support the point in previous studies that thyroid autoantibodies affected fetal growth (29, 31). The mechanism of lower birthweight and higher risk of fetal adverse outcomes is not fully clarified, but we learn from one report that placenta weight is lower in TAI group (19). Thus, parameters of fetal growth and development, as well as maternal nutrition supplement should be aware in pregnant women with TAI.

To better understanding the difference between TAI women and their control individuals, we describe the evolution of maternal TSH, FT4 and FT3 throughout pregnancy base on this large multi-center cohort study. Our result shown that TSH concentrations were higher in TAI women in baseline and remain higher before the third trimester. TAI pregnancies also have higher probability of subclinical hypothyroidism, which is consistent with previous studies (21). Higher FT4 concentration in the TAI group seems to be controversial with elevated TSH concentrations. However, the lower concentration of FT3 in TAI group may explain this phenomenon, since the negative feedback efficiency of FT3 was stronger than FT4 (36). Increase level of both FT4 and TSH was also reported in previous study (37). The potential reason of high FT4 in TAI group might because of the supplementation of levothyroxine in TAI women, but the detail information was not able to obtained in the present retrospective study. As biologically active hormone, FT3 was not elevated sync with FT4 maybe the potential reasons that levothyroxine supplement did not improve pregnancy outcomes in TAI women in previous study (11, 12). To determine the reason of higher FT4 and lower FT3 concentration in TAI pregnant women, further study is needed to study the impact of thyroid antibodies on deiodinase.

There were several strengths of the present study. First, we used a large cohort of pregnant women from three centers in our study to obtain robust results. To the best of our knowledge, the number of participants in this original cohort study was largest on the topic of TAI and pregnancy complications. And based on this cohort, we were able to analyze a number of complications and adverse outcomes both maternal and fetal-neonates by adjusting multiple cofounders. Second, the quantitative association of thyroid autoantibody with pregnancy outcomes was seldom reported. Our result of their dose-dependent association adds to the limited knowledge on the complicated and multifactorial mechanisms underlying pregnancy outcomes. Third, we described the evolution of maternal TSH, FT4 and FT3 in TAI pregnancies and their variation by increasing thyroid autoantibody level. This result may provide insights toward better understanding the difference between TAI women and their control individuals.

Our study also had some limitation. First, the medicine history of levothyroxine (LT-4) supplementation was not obtained due to the retrospective design, and thus the impact of LT-4 supplementation on pregnancy complications were not able to analyze in the present study. Second, although consist of a large number of pregnant women from three centers, the present study is performed in South China where is iodine rich area, and may not represent pregnant women in general population. Third, the underlining mechanism of thyroid autoantibodies and complications were not analyzed in this study. Considering the high prevalence and clinical significance of TAI in pregnancy, further study is needed to determine how thyroid autoantibodies affect related complications.

## Conclusion

We illustrated the independent association between TAI and adverse pregnancy outcomes, including PIH and GDM. We also found neonates born to women with TAI were with lower birthweight and at higher risk for NICU admission. The quantitative association found in the present study between TPOAb and PIH, and between TGAb and PIH, SGA and NICU admission indicates that the dose-dependent effect of thyroid autoimmunity on pregnancy complications should be taken into account in future research and clinical practice.

## Data availability statement

The raw data supporting the conclusions of this article will be made available by the authors, without undue reservation.

## Ethics statement

The studies involving human participants were reviewed and approved by Medical Ethics Committee, Sun Yat-sen Memorial Hospital, Sun Yat-sen University. Written informed consent for participation was not required for this study in accordance with the national legislation and the institutional requirements.

## Author contributions

YX designed the study, analyzed the data and drafted the manuscript. HC and YG provided the clinical data. MR, KS and DL critical review of the study design. HW and RD provided laboratory data. JW and ZL provided clinical data. ZW and LY contributed to the design and critically reviewed the manuscript. All authors contributed to the article and approved the submitted version.

## References

[1] Lee SY Pearce EN. Assessment and treatment of thyroid disorders in pregnancy and the postpartum period. Nat Rev Endocrinol (2022) 18(3):158–71. doi: 10.1038/s41574-021-00604-z PMC9020832 34983968

[2] Krassas GE Poppe K Glinoer D. Thyroid function and human reproductive health. Endocr Rev (2010) 31(5):702–55. doi: 10.1210/er.2009-0041 20573783

[3] Poppe K Velkeniers B Glinoer D. The role of thyroid autoimmunity in fertility and pregnancy. Nat Clin Pract Endocrinol Metab (2008) 4(7):394–405. doi: 10.1038/ncpendmet0846 18506157

[4] Busnelli A Paffoni A Fedele L Somigliana E. The impact of thyroid autoimmunity on IVF/ICSI outcome: a systematic review and meta-analysis. Hum Reprod Update (2016) 22(6):775–90. doi: 10.1093/humupd/dmw019 27323769

[5] McLeod DS Cooper DS. The incidence and prevalence of thyroid autoimmunity. Endocrine (2012) 42(2):252–65. doi: 10.1007/s12020-012-9703-2 22644837

[6] De Leo S Pearce EN. Autoimmune thyroid disease during pregnancy. Lancet Diabetes Endocrinol (2018) 6(7):575–86. doi: 10.1016/S2213-8587(17)30402-3 29246752

[7] Chaker L Razvi S Bensenor IM Azizi F Pearce EN Peeters RP. Hypothyroidism. Nat Rev Dis Primers (2022) 8(1):30. doi: 10.1038/s41572-022-00357-7 35589725

[8] Alexander EK Pearce EN Brent GA Brown RS Chen H Dosiou C. 2017 Guidelines of the American thyroid association for the diagnosis and management of thyroid disease during pregnancy and the postpartum. Thyroid (2017) 27(3):315–89. doi: 10.1089/thy.2016.0457 28056690

[9] Korevaar TIM. Euthyroid thyroperoxidase antibody positivity during pregnancy, to treat or not to treat? Endocrinol Metab (Seoul) (2022) 37(3):387–91. doi: 10.3803/EnM.2022.301 PMC9262680 35798546

[10] Thangaratinam S Tan A Knox E Kilby MD Franklyn J Coomarasamy A. Association between thyroid autoantibodies and miscarriage and preterm birth: meta-analysis of evidence. BMJ (2011) 342:d2616. doi: 10.1136/bmj.d2616 21558126 PMC3089879

[11] Dhillon-Smith RK Middleton LJ Sunner KK Cheed V Baker K Farrell-Carver S. Levothyroxine in women with thyroid peroxidase antibodies before conception. N Engl J Med (2019) 380(14):1316–25. doi: 10.1056/NEJMoa1812537 30907987

[12] van Dijk MM Vissenberg R Fliers E van der Post JAM van der Hoorn MP de Weerd S. Levothyroxine in euthyroid thyroid peroxidase antibody positive women with recurrent pregnancy loss (T4LIFE trial): a multicentre, randomised, double-blind, placebo-controlled, phase 3 trial. Lancet Diabetes Endocrinol (2022) 10(5):322–9. doi: 10.1016/S2213-8587(22)00045-6 35298917

[13] Liu H Shan Z Li C Mao J Xie X Wang W. Maternal subclinical hypothyroidism, thyroid autoimmunity, and the risk of miscarriage: a prospective cohort study. Thyroid (2014) 24(11):1642–9. doi: 10.1089/thy.2014.0029 PMC4229690 25087688

[14] Negro R Formoso G Mangieri T Pezzarossa A Dazzi D Hassan H. Levothyroxine treatment in euthyroid pregnant women with autoimmune thyroid disease: effects on obstetrical complications. J Clin Endocrinol Metab (2006) 91(7):2587–91. doi: 10.1210/jc.2005-1603 16621910

[15] Han Y Mao LJ Ge X Huang K Yan SQ Ren LL. Thyroid autoantibodies in pregnancy are associated with hypertensive disorders of pregnancy: Ma’anshan birth cohort study. Clin Endocrinol (Oxf) (2018) 88(6):928–35. doi: 10.1111/cen.13590 29504633

[16] Abbassi-Ghanavati M Casey BM Spong CY McIntire DD Halvorson LM Cunningham FG. Pregnancy outcomes in women with thyroid peroxidase antibodies. Obstet Gynecol (2010) 116(2 Pt 1):381–6. doi: 10.1097/AOG.0b013e3181e904e5 20664399

[17] Tang L Li P Zhou H Li L. A longitudinal study of thyroid markers during pregnancy and the risk of gestational diabetes mellitus and post-partum glucose metabolism. Diabetes Metab Res Rev (2021) 37(4):e3441. doi: 10.1002/dmrr.3441 33486811 PMC8243952

[18] Ying H Tang YP Bao YR Su XJ Cai X Li YH. Maternal TSH level and TPOAb status in early pregnancy and their relationship to the risk of gestational diabetes mellitus. Endocrine (2016) 54(3):742–50. doi: 10.1007/s12020-016-1022-6 27423217

[19] Mannisto T Vaarasmaki M Pouta A Hartikainen AL Ruokonen A Surcel HM. Perinatal outcome of children born to mothers with thyroid dysfunction or antibodies: a prospective population-based cohort study. J Clin Endocrinol Metab (2009) 94(3):772–9. doi: 10.1210/jc.2008-1520 19106271

[20] Ushijima J Furukawa S Sameshima H. The presence of thyroid peroxidase antibody is associated with lower placental weight in maternal thyroid dysfunction. Tohoku J Exp Med (2019) 249(3):231–6. doi: 10.1620/tjem.249.231 31776300

[21] Bliddal S Derakhshan A Xiao Y Chen LM Mannisto T Ashoor G. Association of thyroid peroxidase antibodies and thyroglobulin antibodies with thyroid function in pregnancy: An individual participant data meta-analysis. Thyroid (2022) 32(7):828–40. doi: 10.1089/thy.2022.0083 35596568

[22] International Association of D Pregnancy Study Groups Consensus P Metzger BE Gabbe SG Persson B Buchanan TA. International association of diabetes and pregnancy study groups recommendations on the diagnosis and classification of hyperglycemia in pregnancy. Diabetes Care (2010) 33(3):676–82. doi: 10.2337/dc09-1848 PMC2827530 20190296

[23] Roberts MJ August AP Bakris G Barton RJ Bernstein MI Druzin M. Hypertension in pregnancy. report of the American college of obstetricians and gynecologists’ task force on hypertension in pregnancy. Obstet Gynecol (2013) 122(5):1122–31. doi: 10.1097/01.AOG.0000437382.03963.88 24150027

[24] Buck Louis GM Grewal J Albert PS Sciscione A Wing DA Grobman WA. Racial/ethnic standards for fetal growth: the NICHD fetal growth studies. Am J Obstet Gynecol (2015) 213(4):449 e1– e41. doi: 10.1016/j.ajog.2015.08.032 PMC4584427 26410205

[25] Lejeune B Grun JP de Nayer P Servais G Glinoer D. Antithyroid antibodies underlying thyroid abnormalities and miscarriage or pregnancy induced hypertension. Br J Obstet Gynaecol (1993) 100(7):669–72. doi: 10.1111/j.1471-0528.1993.tb14236.x 8369252

[26] Chen LM Du WJ Dai J Zhang Q Si GX Yang H. Effects of subclinical hypothyroidism on maternal and perinatal outcomes during pregnancy: a single-center cohort study of a Chinese population. PloS One (2014) 9(10):e109364. doi: 10.1371/journal.pone.0109364 25353960 PMC4212915

[27] Medici M Korevaar TI Schalekamp-Timmermans S Gaillard R de Rijke YB Visser WE. Maternal early-pregnancy thyroid function is associated with subsequent hypertensive disorders of pregnancy: the generation r study. J Clin Endocrinol Metab (2014) 99(12):E2591–8. doi: 10.1210/jc.2014-1505 25157540

[28] Saki F Dabbaghmanesh MH Ghaemi SZ Forouhari S Omrani GR Bakhshayeshkaram M. Thyroid autoimmunity in pregnancy and its influences on maternal and fetal outcome in Iran (a prospective study). Endocr Res (2015) 40(3):139–45. doi: 10.3109/07435800.2014.966384 25330412

[29] Chen LM Zhang Q Si GX Chen QS Ye EL Yu LC. Associations between thyroid autoantibody status and abnormal pregnancy outcomes in euthyroid women. Endocrine (2015) 48(3):924–8. doi: 10.1007/s12020-014-0420-x 25209893

[30] Huang K Xu Y Yan S Li T Xu Y Zhu P. Isolated effect of maternal thyroid-stimulating hormone, free thyroxine and antithyroid peroxidase antibodies in early pregnancy on gestational diabetes mellitus: a birth cohort study in China. Endocr J (2019) 66(3):223–31. doi: 10.1507/endocrj.EJ18-0340 30674732

[31] Karakosta P Alegakis D Georgiou V Roumeliotaki T Fthenou E Vassilaki M. Thyroid dysfunction and autoantibodies in early pregnancy are associated with increased risk of gestational diabetes and adverse birth outcomes. J Clin Endocrinol Metab (2012) 97(12):4464–72. doi: 10.1210/jc.2012-2540 23015651

[32] Negro R Schwartz A Gismondi R Tinelli A Mangieri T Stagnaro-Green A. Thyroid antibody positivity in the first trimester of pregnancy is associated with negative pregnancy outcomes. J Clin Endocrinol Metab (2011) 96(6):E920–4. doi: 10.1210/jc.2011-0026 21411559

[33] Han Y Mao LJ Ge X Huang K Yan SQ Ren LL. Impact of maternal thyroid autoantibodies positivity on the risk of early term birth: Ma’anshan birth cohort study. Endocrine (2018) 60(2):329–38. doi: 10.1007/s12020-018-1576-6 29569122

[34] Stagnaro-Green A Chen X Bogden JD Davies TF Scholl TO. The thyroid and pregnancy: a novel risk factor for very preterm delivery. Thyroid (2005) 15(4):351–7. doi: 10.1089/thy.2005.15.351 15876159

[35] Consortium on T Pregnancy-Study Group on Preterm B Korevaar TIM Derakhshan A Taylor PN Meima M. Association of thyroid function test abnormalities and thyroid autoimmunity with preterm birth: A systematic review and meta-analysis. JAMA (2019) 322(7):632–41. doi: 10.1001/jama.2019.10931 PMC6704759 31429897

[36] Sawin CT Hershman JM Chopra IJ. The comparative effect of T4 and T3 on the TSH response to TRH in young adult men. J Clin Endocrinol Metab (1977) 44(2):273–8. doi: 10.1210/jcem-44-2-273 402379

[37] Williams FL Watson J Ogston SA Visser TJ Hume R Willatts P. Maternal and umbilical cord levels of T4, FT4, TSH, TPOAb, and TgAb in term infants and neurodevelopmental outcome at 5.5 years. J Clin Endocrinol Metab (2013) 98(2):829–38. doi: 10.1210/jc.2012-3572 23322817

