# Supplementary material for: Thyroid autoimmunity and adverse pregnancy outcomes: A multiple center retrospective study
Source: Front Endocrinol (Lausanne). 2023 Feb 27;14:1081851. doi: 10.3389/fendo.2023.1081851 (PMC10008944; doi:10.3389/fendo.2023.1081851)
Supplement: Supplementary file 1 [file DataSheet_1.pdf]

Table S1 Laboratory Conditions and Reference Range of The Kits

	Abbott[1]		Beckman[2]		Siemens[3]	
	**Lower range**	**Upper range**	**Lower range**	**Upper range**	**Lower range**	**Upper range**
TPOAb (U/L)	0	5.61	0	9.00	0	60.00
TGAb (U/L)	0	4.11	0	3.99	0	60.00
TSH (μIU/ml)						
Non-Pregnant	0.35	4.94	0.56	5.91	0.55	4.78
First Trimester	0.07	3.38	0.05	3.55	0.08	3.00
Second Trimester	0.34	3.51	0.21	3.31	0.31	2.97
Third Trimester	0.34	4.32	0.43	3.71	0.45	4.95
Free T4 (pmol/L)						
Non-Pregnant	9.01	19.01	7.5	21.1	11.50	22.70
First Trimester	11.30	17.80	9.01	15.89	11.30	18.32
Second Trimester	9.30	15.20	6.62	13.51	9.49	14.19
Third Trimester	7.90	14.10	6.42	10.75	8.51	13.93
Free T3 (pmol/L)						
Non-Pregnant	2.43	6.01	3.81	6.91	3.5	6.5

The reference range of non-pregnant status was according to the instruction manuals of each kit.
The reference ranges of pregnant period were according to the report of Chinese guideline. DOI:10.3760/cma.j.issn.1000-6699.2019.08.003.

1. ARCHITECT i2000SR immunoassay analyzer (Abbott Laboratories, Singapore). The intra-assay coefficients of variation (CV) of serum TSH, free T4, free T3, TPOAb, and TGAb were 1.1%-5.0%, 2.3%-5.3%,1.4%-4.2%,1.8%-9.5% and 1.7%-6.6% respectively. The inter-assay CV values were 1.7%-5.3%, 3.6%-7.8%, 2.3%-4.6%, 2.5%-9.8% and 2.3%-8.2% respectively.
2. UniCel Dxi 800 Immunoassay System (Beckman Coulter, USA). The intra-assay CV of serum TSH, free T4, free T3, TPOAb, and TGAb were 2.0%-4.0%, 1.8%-4.4%, 2.6%-6.6%, 5.1%-7.1% and 3.6%-5.7% respectively. The inter-assay CV values were 0.2%-2%, 3.34%-8.08%, 1.3%-8.0%, 2.2%-4.4% and 0%-7.7% respectively.
3. Atellica IM 1600 Analyzer (Siemens Healthcare Diagnostics, USA). The intra-assay CV of serum TSH, free T4, free T3, TPOAb, and TGAb were 1.2%-7.0%,0.13%-3.48%, 0.05%-0.49%, 1.5%-4.1% and 1.3%-5.0% respectively. The inter-assay CV values were 2.6%-8.5%, 0.39%-4.77%, 1.07%-9.14%, 3.4%-7.3% and 3.0%-6.7% respectively.

Table S2 TPOAb and TGAb Concentrations in Participants with and Without Maternal and Fetal-neonatal Complications

	TPOAb			TGAb		
	**Case**	**Control**	***P value***	**Case**	**Control**	***P value***
**Maternal Outcomes**						
Pregnancy Induce Hypertension	0.519 ±0.292	0.499 ±0.286	**0.038**	0.522 ±0.284	0.499 ±0.283	**0.030**
Gestational Diabetes Mellitus	0.507 ±0.289	0.498 ±0.285	**0.033**	0.496 ±0.281	0.501 ±0.284	0.290
Preterm Birth	0.517 ±0.284	0.498 ±0.286	**0.003**	0.516 ±0.281	0.499 ±0.284	**0.020**
Premature Rupture of Membrane	0.496 ±0.286	0.501 ±0.286	0.299	0.495 ±0.285	0.501 ±0.283	0.183
Postpartum Hemorrhage	0.498 ±0.286	0.500 ±0.286	0.709	0.495 ±0.284	0.501 ±0.284	0.415
**Fetal-neonatal Outcomes**						
NICU Admission	0.505 ±0.287	0.498 ±0.285	0.099	0.508 ±0.283	0.498 ±0.284	**0.045**
Low Apgar score	0.495 ±0.282	0.500 ±0.286	0.670	0.481 ±0.279	0.500 ±0.284	0.109
Macrosomia	0.476 ±0.280	0.501 ±0.286	**0.042**	0.492 ±0.279	0.500 ±0.284	0.550
Large for Gestational Age	0.488 ±0.280	0.500 ±0.286	0.190	0.491 ±0.281	0.500 ±0.283	0.388
Small for Gestational Age	0.510 ±0.287	0.500 ±0.286	0.085	0.514 ±0.287	0.500 ±0.283	**0.032**

Data are population-based percentiles of concentrations (mean ± SD)
